# Otologic Manifestations and Peripheral Facial Nerve Palsy As the Initial Presentation of Granulomatosis With Polyangiitis: A Case Report

**DOI:** 10.7759/cureus.114821

**Published:** 2026-08-19

**Authors:** Alex Hamdar, Reda Chelly, Liza Charlier, Ngongang Ouandji A David, Clément Lelong

**Affiliations:** 1 Otolaryngology - Head and Neck Surgery, Hôpital Universitaire de Bruxelles (HUB) Erasme Hospital, Brussels, BEL

**Keywords:** anca-associated vasculitis, facial nerve palsy, granulomatosis with polyangiitis, hearing loss, labyrinthitis, refractory otitis media

## Abstract

Granulomatosis with polyangiitis (GPA) is a rare antineutrophil cytoplasmic antibody (ANCA)-associated vasculitis that predominantly affects the upper and lower respiratory tracts and the kidneys. Although otolaryngological manifestations are common, isolated otologic symptoms as the initial presentation are rare and may delay diagnosis. We report the case of a 65-year-old man who initially presented with refractory bilateral otitis media associated with progressive mixed hearing loss, vestibular dysfunction, and subsequent peripheral facial nerve palsy. Initial temporal bone imaging was unremarkable, showing only middle ear and mastoid effusion. Further investigations revealed ANCA positivity and bilateral necrotizing pulmonary opacities on chest CT, leading to the diagnosis of GPA. Treatment with rituximab and corticosteroids resulted in marked improvement of hearing, vestibular symptoms, and facial nerve function. This case highlights the importance of considering GPA in patients with refractory otologic disease, particularly when associated with severe otalgia, sensorineural hearing loss, vertigo, or facial nerve palsy. Early recognition of these atypical manifestations and prompt multidisciplinary management are essential to prevent irreversible organ damage and improve clinical outcomes.

## Introduction

Granulomatosis with polyangiitis (GPA), formerly known as Wegener's granulomatosis, is a rare autoimmune disease characterized by granulomatous inflammation and necrotizing vasculitis predominantly affecting small- and medium-sized blood vessels [1,2]. Although uncommon, GPA is well recognized because of its potentially severe multisystem involvement. The complexity of its diagnosis and management requires a multidisciplinary approach involving otolaryngologists, internists, nephrologists, and pulmonologists [3].

Although GPA can affect virtually any organ, it predominantly involves the upper and lower respiratory tracts and the kidneys [1,4]. Otorhinolaryngological (ENT) manifestations occur in approximately 80-95% of patients during the course of the disease [1,5]. GPA typically shows a bimodal age distribution, with most cases diagnosed between 40 and 60 years of age [2,6].

Within the spectrum of ENT manifestations, otologic involvement is considerably less common than sinonasal disease. However, it may represent the initial or even the sole manifestation of GPA, frequently mimicking common infectious conditions [2,7]. Clinical manifestations include otitis media, sensorineural hearing loss, vestibular dysfunction resembling acute labyrinthitis, and, more rarely, peripheral facial nerve palsy. Early recognition of these otologic "red flags" by otolaryngologists is essential, as delayed diagnosis may lead to rapid progression toward life-threatening pulmonary disease or irreversible renal failure [1,6].

We report the case of a 65-year-old man presenting with an atypical course of otitis media that ultimately led to the diagnosis of GPA. This case highlights the importance of considering systemic vasculitis in patients with refractory otologic disease to facilitate early diagnosis and prompt initiation of immunosuppressive therapy.

## Case presentation

A 65-year-old man with no significant past medical history presented to the ENT clinic with bilateral otalgia and a sensation of ear fullness. One month earlier, he had experienced an episode of rhinosinusitis, for which a course of antibiotics prescribed by his primary care physician had been ineffective.

On otomicroscopy, acute otitis media with bullous myringitis was observed on the left side, while otitis media with effusion was present on the right. Nasal endoscopy was unremarkable. Pure-tone audiometry demonstrated bilateral mixed hearing loss (Figure 1). Second-line antibiotic therapy, a short course of systemic corticosteroids, and local nasal care were initiated.

**Figure 1:**
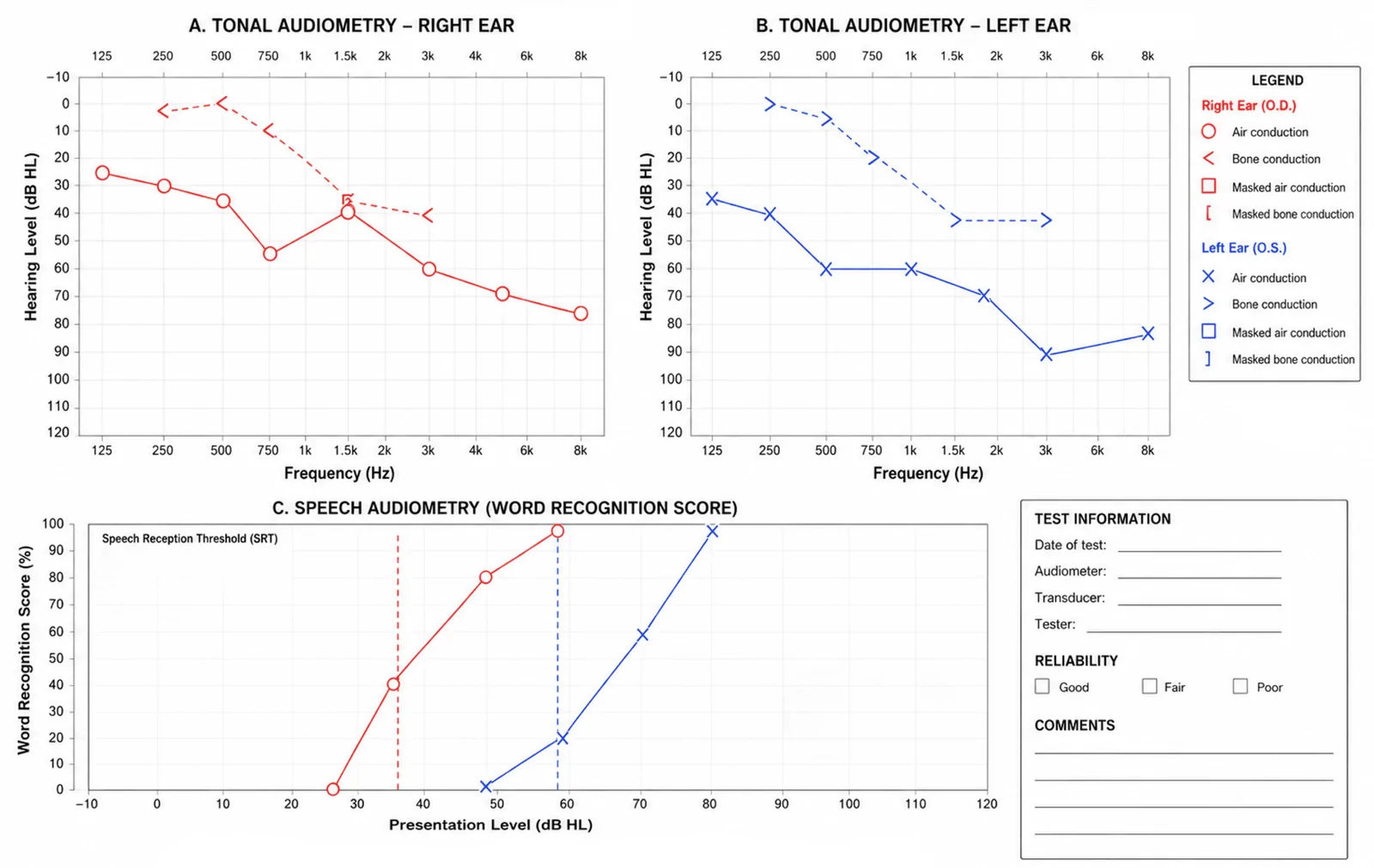
Initial audiological assessment. Pure-tone audiometry of the right ear (A) and left ear (B), with corresponding speech audiometry (C), demonstrating bilateral mixed hearing loss dB HL: hearing level in decibels, HL reference: ANSI S3.6/ISO 389, lower on the graph: poorer hearing, 0 dB HL: normal hearing level (young adults)

After one week of appropriate treatment, the patient reported worsening otalgia and hearing loss. Bilateral myringotomy was therefore performed, yielding purulent fluid from the left ear and seromucous fluid from the right.

One week later, he presented to the emergency department with sudden-onset rotational vertigo accompanied by vomiting. Vestibular examination revealed spontaneous left-beating nystagmus, an abnormal right-sided head impulse test, and worsening mixed hearing loss, predominantly on the right. Right-sided labyrinthitis was suspected.

A tympanostomy tube was placed in the right ear but could not be placed in the left ear because of the associated myringitis (Figure 2). The patient was admitted for observation and continued corticosteroid therapy. Temporal bone CT and MRI demonstrated only fluid opacification of the middle ear and mastoid air cells, without evidence of bone erosion or suspicious lesions (Figures 3-4). Following symptomatic improvement, the patient was discharged from the hospital.

**Figure 2:**
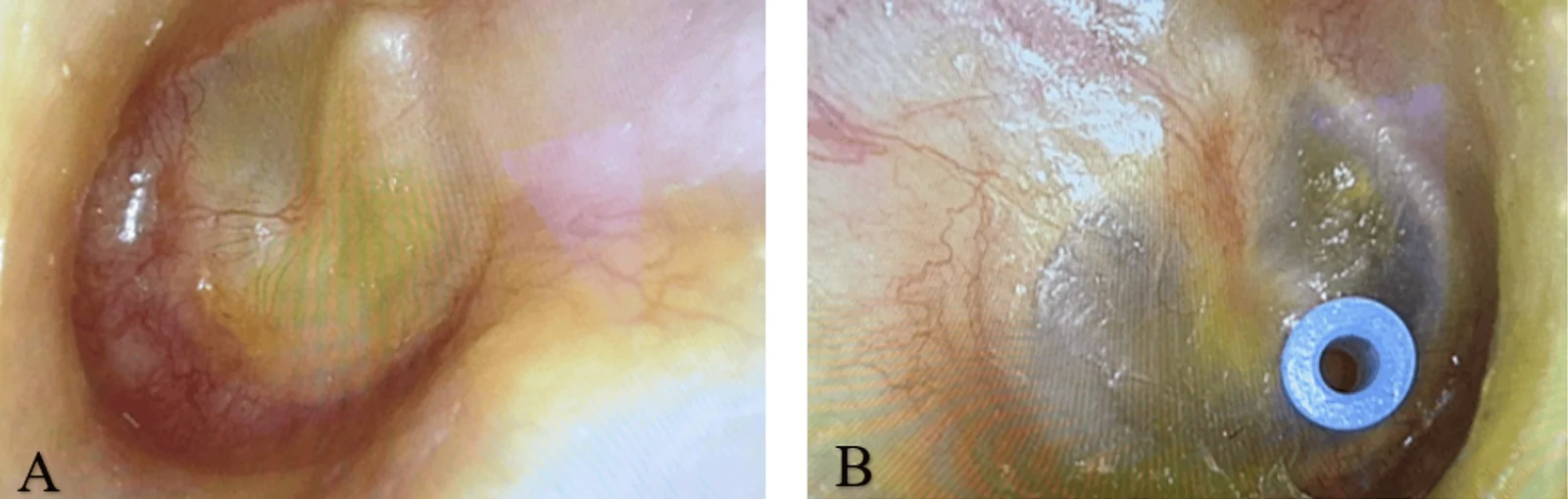
Otomicroscopic examination showing a thickened and inflamed left tympanic membrane (A) and a right tympanic membrane with a tympanostomy tube in place (B)

**Figure 3:**
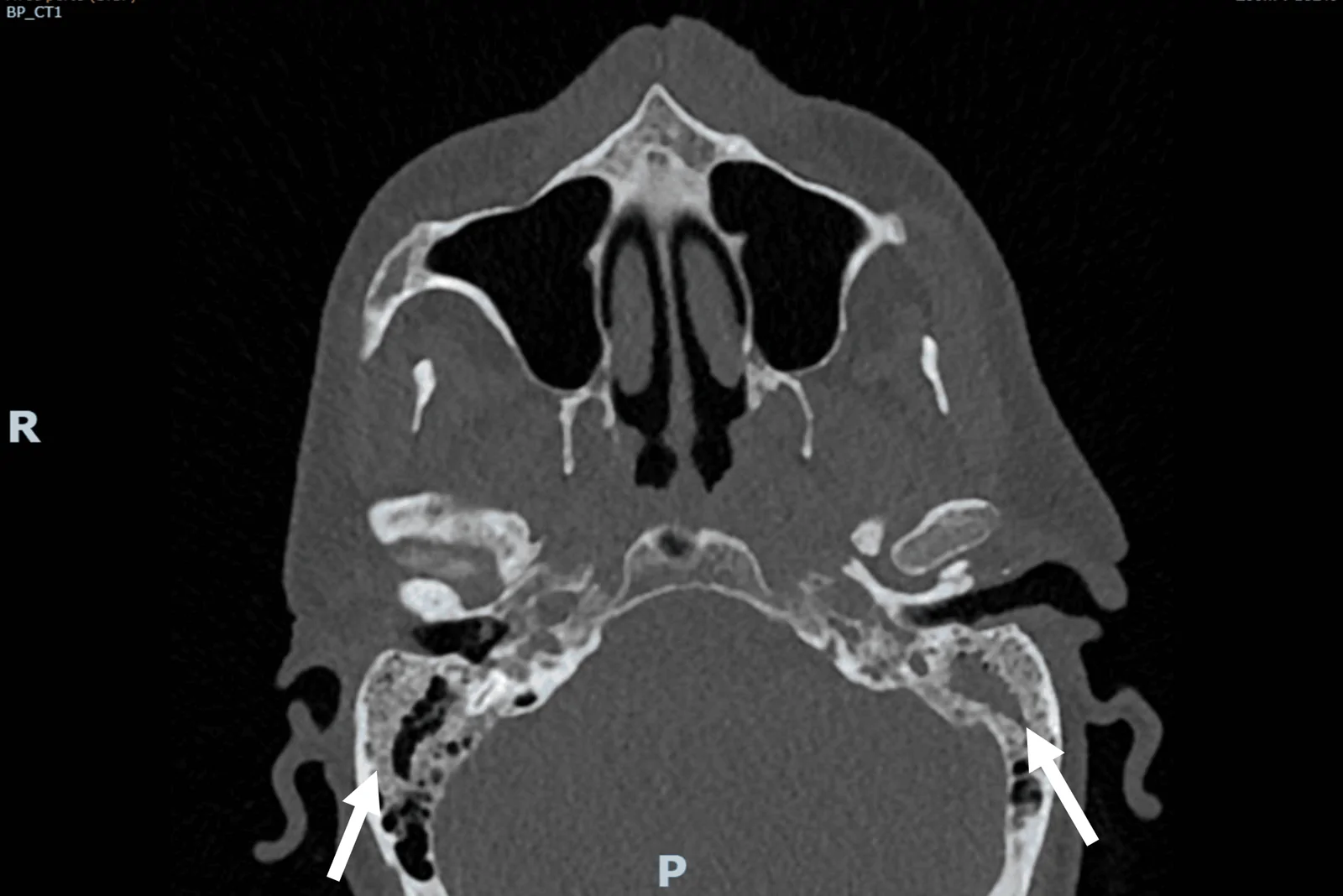
Axial high-resolution CT of the temporal bones showing extensive opacification of the left mastoid air cells (arrow) and middle ear cavity, with extensive opacification of the left mastoid air cells and middle ear cavity and limited involvement of the right anterior mastoid air cells and dependent portion of the right middle ear cavity (arrows) CT: computed tomography

**Figure 4:**
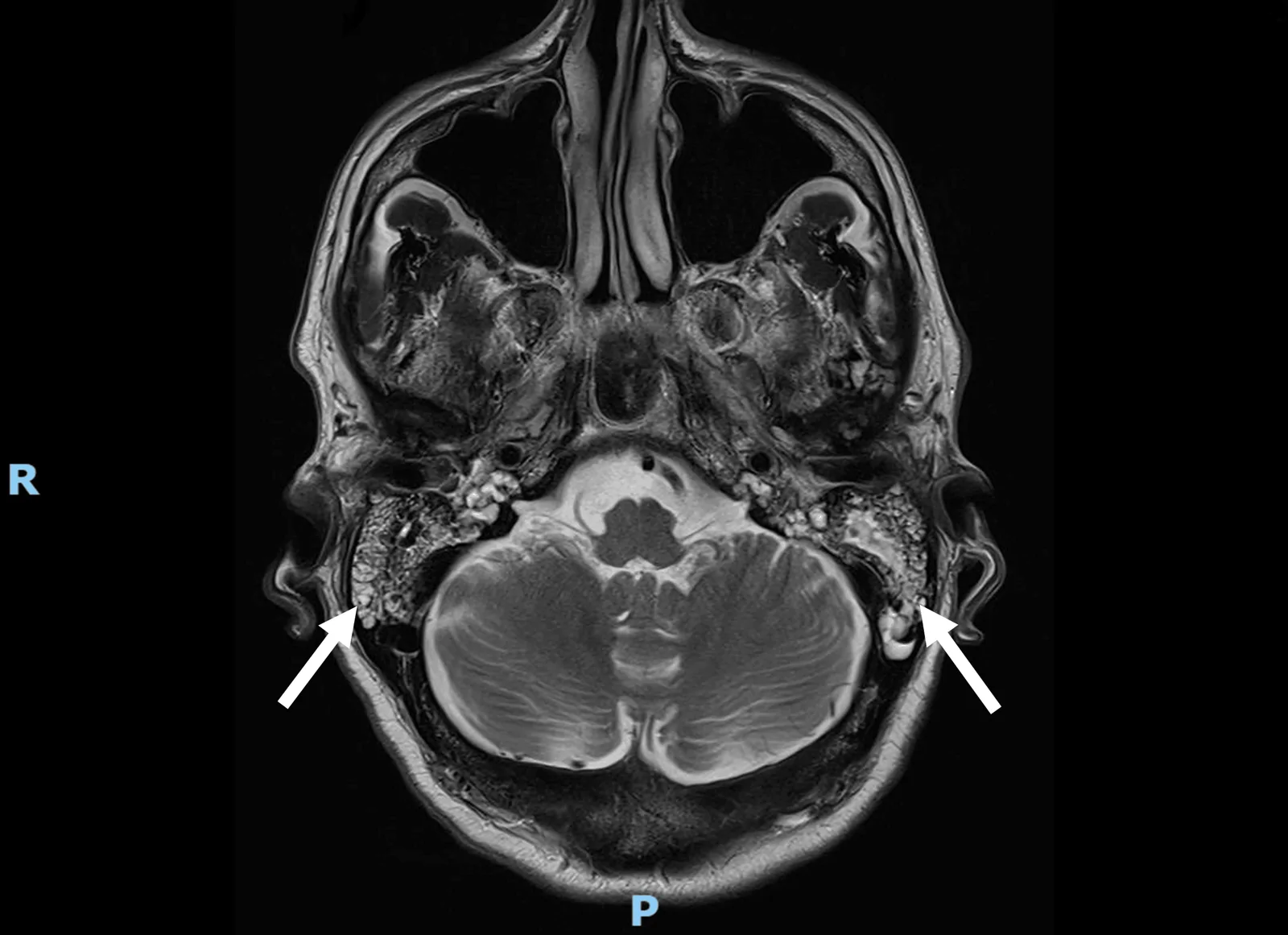
Axial T2-weighted MRI demonstrating bilateral inflammatory soft-tissue/fluid opacification of the middle ear cavities and mastoid air cells (arrows). No evidence of labyrinthitis or intracranial venous thrombosis was identified MRI: magnetic resonance imaging

One week later, the patient was readmitted with a right peripheral facial nerve palsy (House-Brackmann grade III). No herpetic vesicles were observed, and the right tympanostomy tube remained in place with residual seromucous drainage. Given this new neurological deficit in the setting of refractory otitis media, a systemic workup was undertaken.

Blood tests revealed elevated inflammatory markers, with an erythrocyte sedimentation rate of 71 mm/h and a C-reactive protein level of 19.2 mg/L. Renal function was preserved, with a serum creatinine level of 1.10 mg/dL, and urinalysis showed neither proteinuria nor hematuria. Cytoplasmic antineutrophil cytoplasmic antibodies (C-ANCA) were positive, with an anti-proteinase 3 (PR3) antibody level of 155.5 U/mL, whereas anti-myeloperoxidase antibodies were negative. QuantiFERON-TB Gold testing was negative. Viral and bacterial investigations were otherwise negative. Lumbar puncture was performed to exclude meningeal or other neurological involvement. Chest CT demonstrated bilateral bibasilar peribronchovascular pulmonary opacities, some with necrotic features (Figures 5-6).

**Figure 5:**
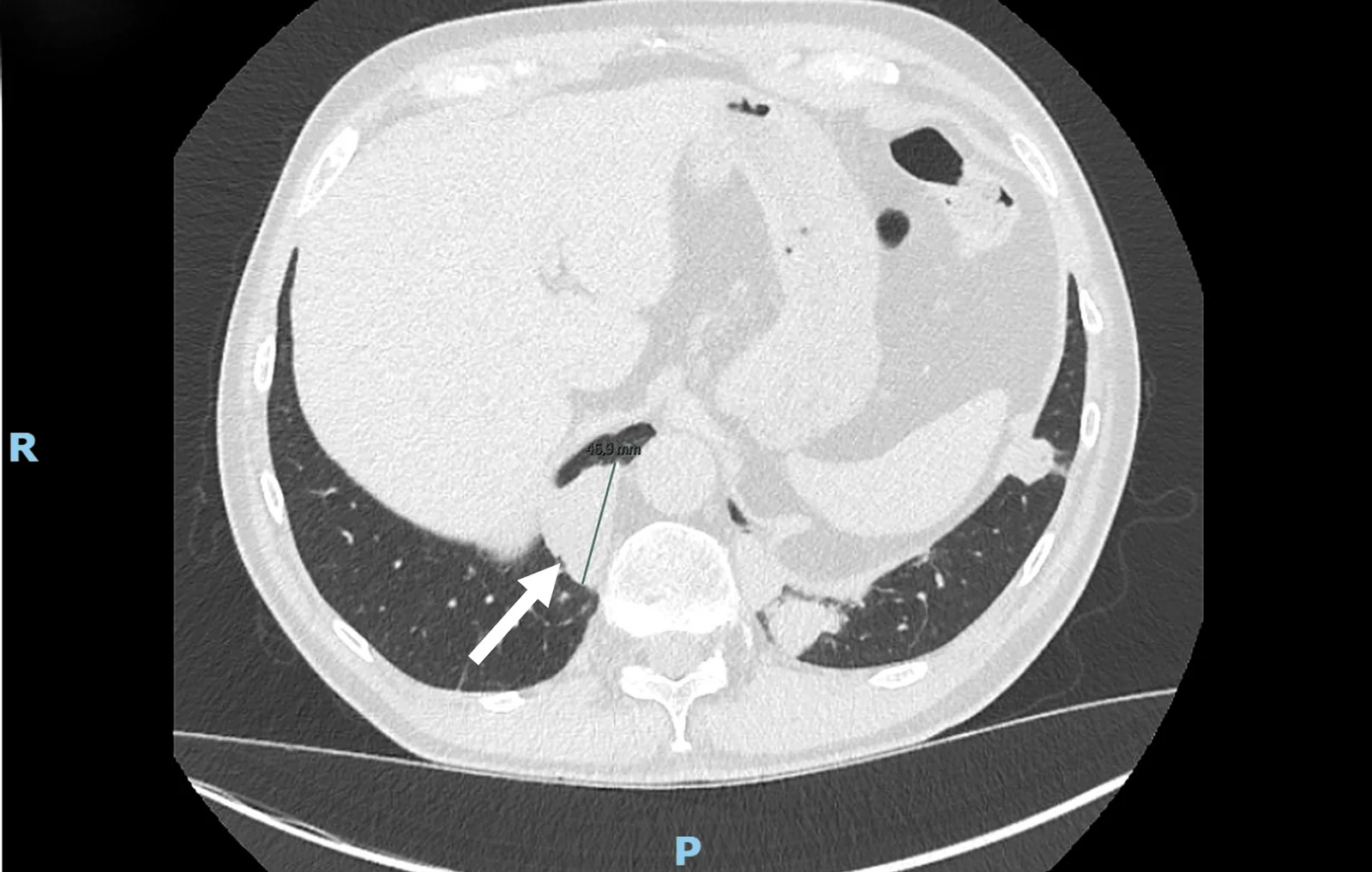
Axial contrast-enhanced chest CT revealing a right lower lobe peribronchovascular pulmonary opacity measuring 47 mm in maximum axial diameter (arrow) CT: computed tomography

**Figure 6:**
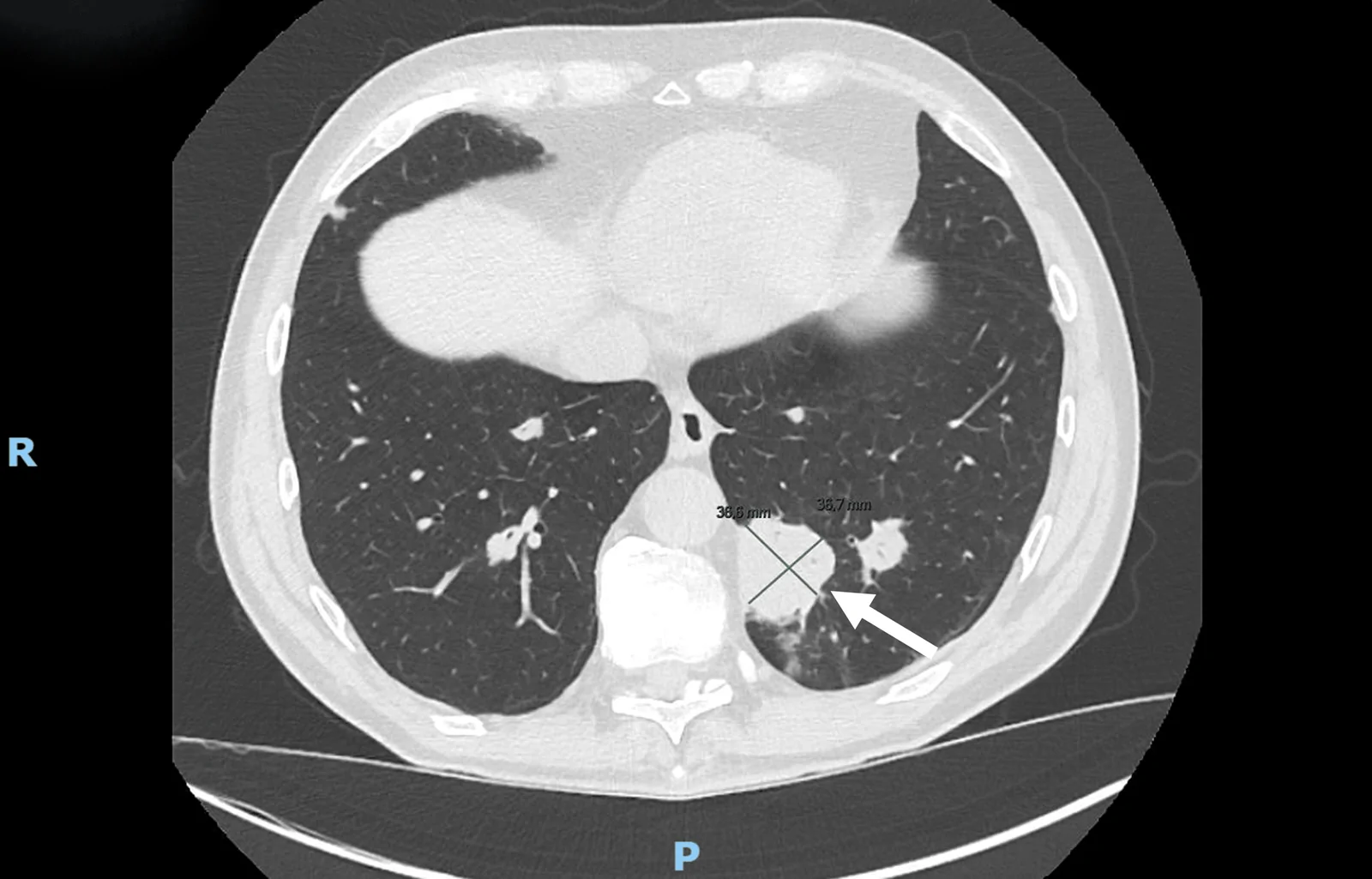
Axial contrast-enhanced chest CT showing a left lobe peribronchovascular pulmonary opacity measuring 37 mm in maximum axial diameter (arrow) CT: computed tomography

The combination of severe otologic involvement (otitis media, clinically suspected labyrinthitis, and facial nerve palsy), necrotic pulmonary lesions, and a positive PR3-ANCA supported the diagnosis of GPA, and the patient met the 2022 ACR/EULAR classification criteria with a score of 7. Histopathological confirmation was not obtained because no accessible nasal or otologic lesion suitable for biopsy was identified.

Treatment with rituximab and a new corticosteroid tapering regimen was initiated, together with facial physiotherapy. Methylprednisolone was administered at 64 mg/day during the first week and 32 mg/day during the second week, with the dose progressively decreased every two weeks thereafter.

After three months of treatment, including three rituximab infusions, the facial nerve palsy had almost completely resolved, and hearing had improved despite persistent mixed hearing loss on pure-tone audiometry and intermittent clear otorrhea through the tympanostomy tube (Figure 7).

**Figure 7:**
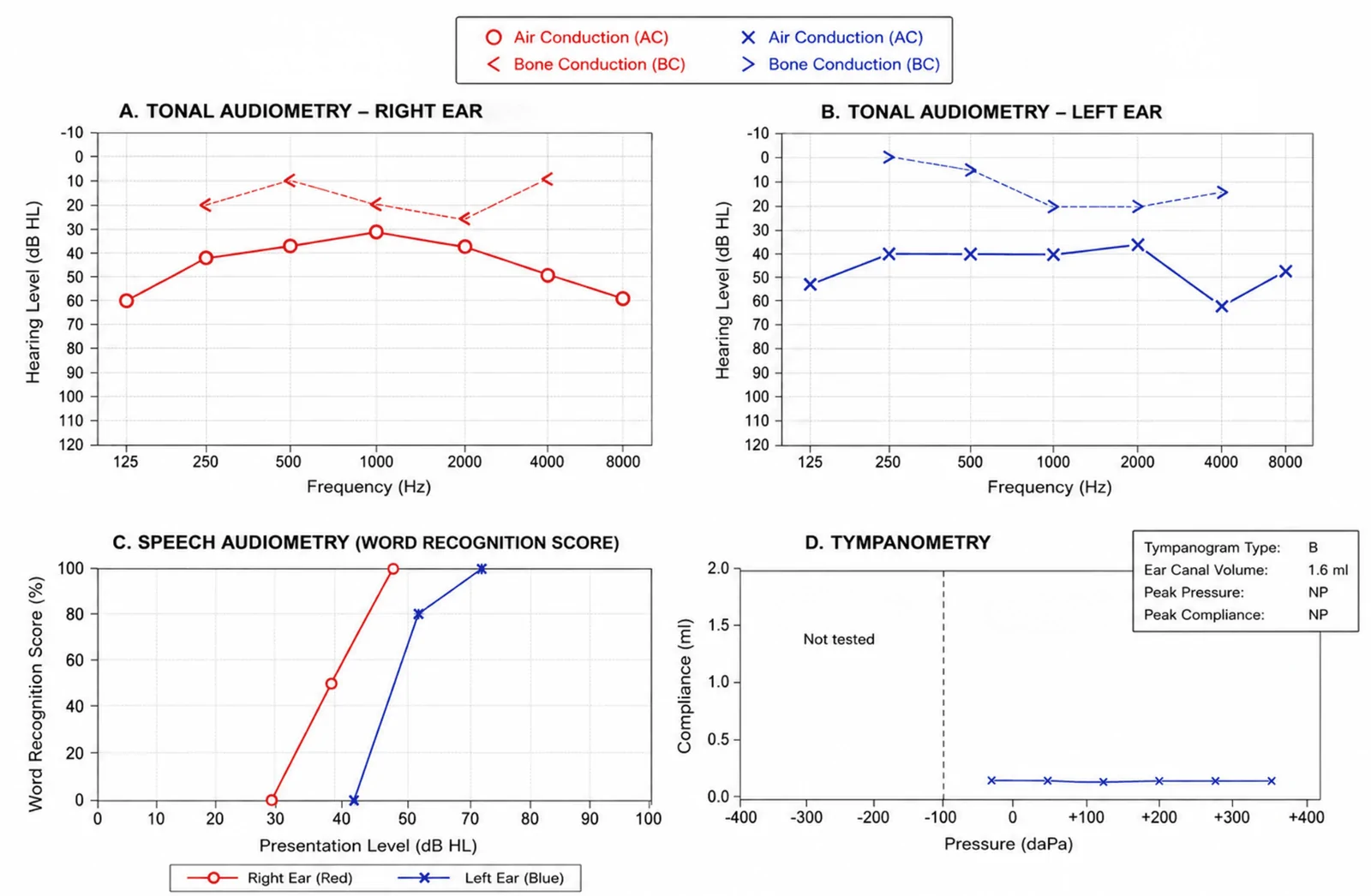
Follow-up audiological assessment three months after initiation of treatment. Pure-tone audiometry of the right ear (A) and left ear (B), with corresponding speech audiometry (C) and tympanometry (D), demonstrating marked improvement in hearing thresholds and speech discrimination compared with the initial evaluation AC: air conduction, BC: bone conduction, dB HL: hearing level in decibels, Hz: Hertz, daPa: decaPascal, NP: no peak

## Discussion

Diagnosing GPA remains a major clinical challenge. Although sinonasal involvement is the most common manifestation (70-90% of cases), it may be absent, as illustrated by our patient [1].

Otologic manifestations, including acute otitis media, otitis media with effusion, sensorineural hearing loss, and vertigo, occur in approximately 20-45% of patients [5,7]. However, they represent the initial and isolated presentation in only 2-10% of cases [2,6]. Facial nerve palsy, whether unilateral or bilateral, is uncommon, occurring in fewer than 5% of patients, and is thought to result either from compression of the facial nerve by granulomatous tissue or from vasculitic involvement of the vasa nervorum [4].

In patients presenting with refractory otitis media, several differential diagnoses should be considered, including persistent acute bacterial otitis media, chronic otitis media with effusion, middle ear tuberculosis, and middle ear neoplasms [7]. Nevertheless, our case suggests that otitis media with effusion associated with recent-onset sensorineural hearing loss or severe otalgia should prompt suspicion of GPA, particularly when accompanied by facial nerve palsy or contralateral ear involvement [2,3].

According to otopathological studies, the mechanisms underlying otologic manifestations are multifactorial [8]. Conductive hearing loss results from inflammation of the middle ear and Eustachian tube, leading to middle ear effusion, whereas sensorineural (or mixed) hearing loss may result from extension of granulomatous inflammation or ischemic injury affecting the cochlear blood supply [4,9]. Vertigo is believed to result from labyrinthine ischemia or hemorrhage involving the anterior vestibular or labyrinthine artery [9]. Facial nerve palsy most likely reflects compression of the facial nerve within the fallopian canal by granulomatous tissue or ischemic injury secondary to vasculitis of the vasa nervorum [1].

In the presence of atypical otologic manifestations, prompt systemic evaluation should be initiated. This evaluation should include (1) a detailed medical history focusing on systemic symptoms such as cough, dyspnea, hematuria, myalgia, and arthralgia; (2) flexible nasal endoscopy to identify asymptomatic nasal lesions, including granulomas, vestibulitis, or septal perforation, with biopsy when appropriate; (3) laboratory investigations, including ANCA testing, recognizing that although PR3-ANCA positivity is highly suggestive of GPA, negative results do not exclude the diagnosis, particularly in limited forms of the disease [2,3]; and (4) imaging studies, especially temporal bone and chest CT, to assess for pulmonary nodules or necrotizing lesions [1]. Despite the systemic nature of the disease, renal involvement was not identified in our patient, with preserved renal function and no hematuria or proteinuria.

Standard induction therapy consists of high-dose corticosteroids in combination with immunosuppressive agents such as rituximab [3,10]. With appropriate treatment, clinical remission is achieved in approximately 75-90% of patients, typically within six months [4,10]. Treatment is generally maintained for 18 months to four years, depending on disease severity and relapse risk. Longer treatment duration is recommended in patients with pulmonary or cardiovascular involvement, persistent PR3-ANCA positivity, or a history of relapse [10]. Without treatment, GPA is associated with a poor prognosis, with a mean survival of less than one year and a two-year survival rate of approximately 10% [1,6].

This case highlights the pivotal role of the otolaryngologist in recognizing atypical presentations of GPA. Early diagnosis and prompt initiation of immunosuppressive therapy are essential to prevent irreversible organ damage and improve patient outcomes.

## Conclusions

This case demonstrates an uncommon initial presentation of GPA. It highlights the importance of considering this diagnosis in patients with refractory otitis media, particularly when associated with severe otalgia, mixed or sensorineural hearing loss, vestibular symptoms, or facial nerve palsy.

Early multidisciplinary management is crucial to prevent irreversible hearing loss and life-threatening systemic complications. Given the high risk of relapse, long-term follow-up remains essential to detect disease recurrence and monitor otolaryngological, pulmonary, and renal involvement.
